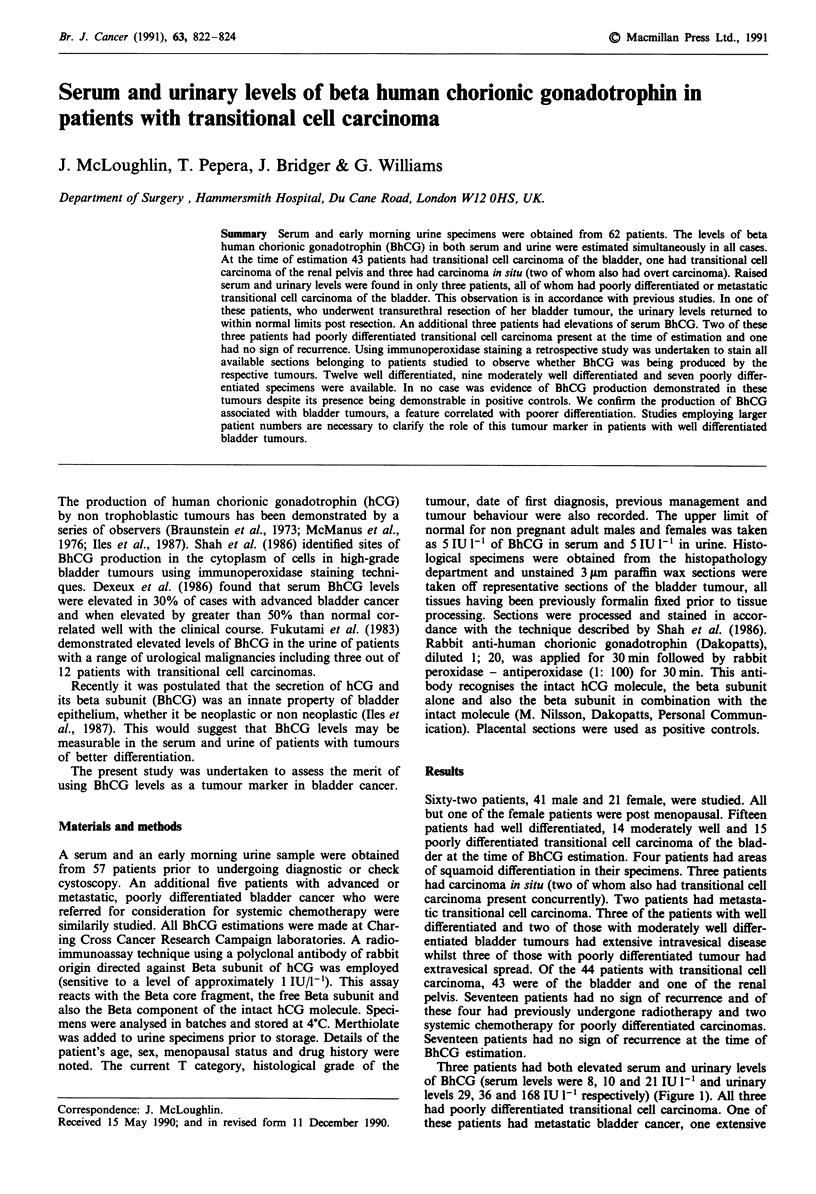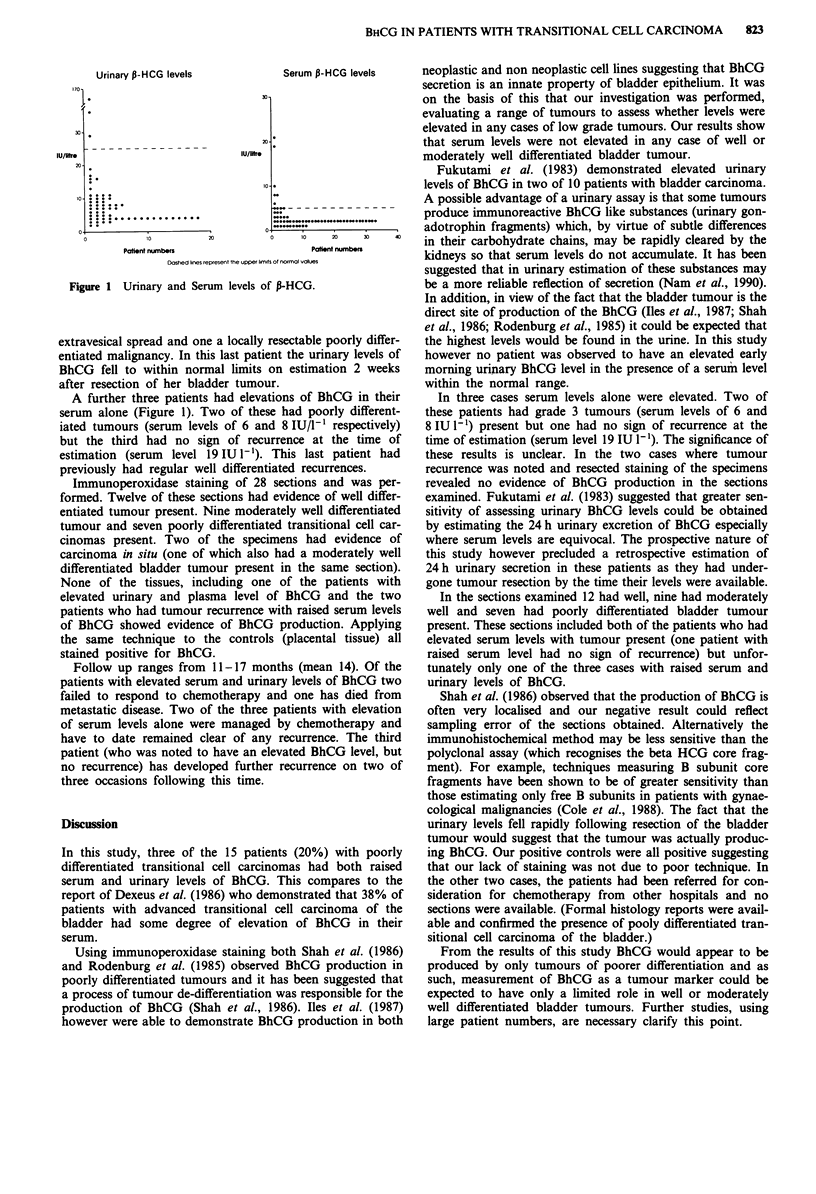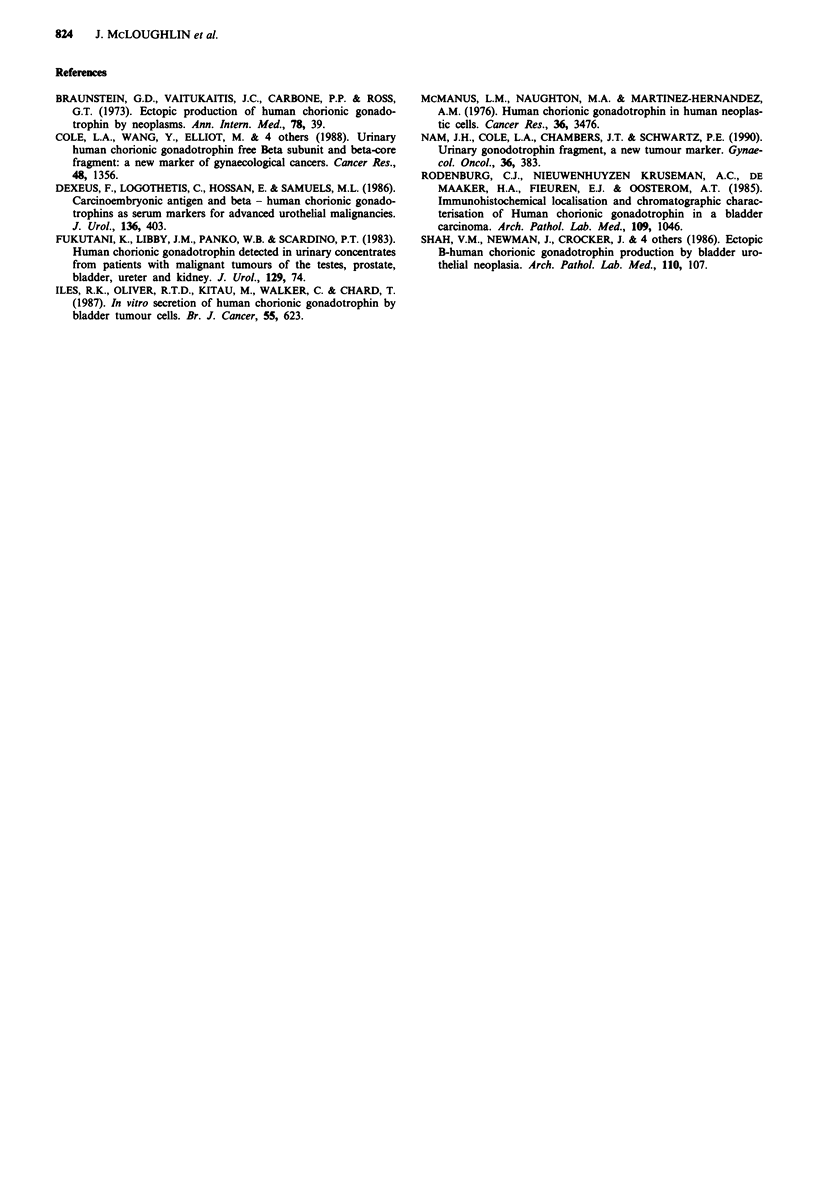# Serum and urinary levels of beta human chorionic gonadotrophin in patients with transitional cell carcinoma.

**DOI:** 10.1038/bjc.1991.182

**Published:** 1991-05

**Authors:** J. McLoughlin, T. Pepera, J. Bridger, G. Williams

**Affiliations:** Department of Surgery, Hammersmith Hospital, London, UK.

## Abstract

Serum and early morning urine specimens were obtained from 62 patients. The levels of beta human chorionic gonadotrophin (BhCG) in both serum and urine were estimated simultaneously in all cases. At the time of estimation 43 patients had transitional cell carcinoma of the bladder, one had transitional cell carcinoma of the renal pelvis and three had carcinoma in situ (two of whom also had overt carcinoma). Raised serum and urinary levels were found in only three patients, all of whom had poorly differentiated or metastatic transitional cell carcinoma of the bladder. This observation is in accordance with previous studies. In one of these patients, who underwent transurethral resection of her bladder tumour, the urinary levels returned to within normal limits post resection. An additional three patients had elevations of serum BhCG. Two of these three patients had poorly differentiated transitional cell carcinoma present at the time of estimation and one had no sign of recurrence. Using immunoperoxidase staining a retrospective study was undertaken to stain all available sections belonging to patients studied to observe whether BhCG was being produced by the respective tumours. Twelve well differentiated, nine moderately well differentiated and seven poorly differentiated specimens were available. In no case was evidence of BhCG production demonstrated in these tumours despite its presence being demonstrable in positive controls. We confirm the production of BhCG associated with bladder tumours, a feature correlated with poorer differentiation. Studies employing larger patient numbers are necessary to clarify the role of this tumor marker in patients with well differentiated bladder tumours.


Br. J. Cancer (1991), 63, 822-824 © Macmillan Press Ltd., 1991

### Serum and urinary levels of beta human chorionic gonadotrophin in patients with transitional cell carcinoma

J. McLoughlin, T. Pepera, J. Bridger & G. Williams

*Department of Surgery, Hammersmith Hospital, Du Cane Road, London W12 0HS, UK.*

**Summary** Serum and early morning urine specimens were obtained from 62 patients. The levels of beta human chorionic gonadotrophin (BhCG) in both serum and urine were estimated simultaneously in all cases. At the time of estimation 43 patients had transitional cell carcinoma of the bladder, one had transitional cell carcinoma of the renal pelvis and three had carcinoma *in situ* (two of whom also had overt carcinoma). Raised serum and urinary levels were found in only three patients, all of whom had poorly differentiated or metastatic transitional cell carcinoma of the bladder. This observation is in accordance with previous studies. In one of these patients, who underwent transurethral resection of her bladder tumour, the urinary levels returned to within normal limits post resection. An additional three patients had elevations of serum BhCG. Two of these three patients had poorly differentiated transitional cell carcinoma present at the time of estimation and one had no sign of recurrence. Using immunoperoxidase staining a retrospective study was undertaken to stain all available sections belonging to patients studied to observe whether BhCG was being produced by the respective tumours. Twelve well differentiated, nine moderately well differentiated and seven poorly differentiated specimens were available. In no case was evidence of BhCG production demonstrated in these tumours despite its presence being demonstrable in positive controls. We confirm the production of BhCG associated with bladder tumours, a feature correlated with poorer differentiation. Studies employing larger patient numbers are necessary to clarify the role of this tumour marker in patients with well differentiated bladder tumours.

The production of human chorionic gonadotrophin (hCG) by non trophoblastic tumours has been demonstrated by a series of observers (Braunstein *et al.*, 1973; McManus *et al.*, 1976; Iles *et al.*, 1987). Shah *et al.* (1986) identified sites of BhCG production in the cytoplasm of cells in high-grade bladder tumours using immunoperoxidase staining techniques. Dexeux *et al.* (1986) found that serum BhCG levels were elevated in 30% of cases with advanced bladder cancer and when elevated by greater than 50% than normal correlated well with the clinical course. Fukutami *et al.* (1983) demonstrated elevated levels of BhCG in the urine of patients with a range of urological malignancies including three out of 12 patients with transitional cell carcinomas.

Recently it was postulated that the secretion of hCG and its beta subunit (BhCG) was an innate property of bladder epithelium, whether it be neoplastic or non neoplastic (Iles *et al.*, 1987). This would suggest that BhCG levels may be measurable in the serum and urine of patients with tumours of better differentiation.

The present study was undertaken to assess the merit of using BhCG levels as a tumour marker in bladder cancer.

### Materials and methods

A serum and an early morning urine sample were obtained from 57 patients prior to undergoing diagnostic or check cystoscopy. An additional five patients with advanced or metastatic, poorly differentiated bladder cancer who were referred for consideration for systemic chemotherapy were similarily studied. All BhCG estimations were made at Charing Cross Cancer Research Campaign laboratories. A radioimmunoassay technique using a polyclonal antibody of rabbit origin directed against Beta subunit of hCG was employed (sensitive to a level of approximately 1 $IU/l^{-1}$). This assay reacts with the Beta core fragment, the free Beta subunit and also the Beta component of the intact hCG molecule. Specimens were analysed in batches and stored at 4°C. Merthiolate was added to urine specimens prior to storage. Details of the patient's age, sex, menopausal status and drug history were noted. The current T category, histological grade of the tumour, date of first diagnosis, previous management and tumour behaviour were also recorded. The upper limit of normal for non pregnant adult males and females was taken as 5 $IU\,l^{-1}$ of BhCG in serum and 5 $IU\,l^{-1}$ in urine. Histological specimens were obtained from the histopathology department and unstained 3 $\mu m$ paraffin wax sections were taken off representative sections of the bladder tumour, all tissues having been previously formalin fixed prior to tissue processing. Sections were processed and stained in accordance with the technique described by Shah *et al.* (1986). Rabbit anti-human chorionic gonadotrophin (Dakopatts), diluted 1; 20, was applied for 30 min followed by rabbit peroxidase – antiperoxidase (1: 100) for 30 min. This antibody recognises the intact hCG molecule, the beta subunit alone and also the beta subunit in combination with the intact molecule (M. Nilsson, Dakopatts, Personal Communication). Placental sections were used as positive controls.

### Results

Sixty-two patients, 41 male and 21 female, were studied. All but one of the female patients were post menopausal. Fifteen patients had well differentiated, 14 moderately well and 15 poorly differentiated transitional cell carcinoma of the bladder at the time of BhCG estimation. Four patients had areas of squamoid differentiation in their specimens. Three patients had carcinoma *in situ* (two of whom also had transitional cell carcinoma present concurrently). Two patients had metastatic transitional cell carcinoma. Three of the patients with well differentiated and two of those with moderately well differentiated bladder tumours had extensive intravesical disease whilst three of those with poorly differentiated tumour had extravesical spread. Of the 44 patients with transitional cell carcinoma, 43 were of the bladder and one of the renal pelvis. Seventeen patients had no sign of recurrence and of these four had previously undergone radiotherapy and two systemic chemotherapy for poorly differentiated carcinomas. Seventeen patients had no sign of recurrence at the time of BhCG estimation.

Three patients had both elevated serum and urinary levels of BhCG (serum levels were 8, 10 and 21 $IU\,l^{-1}$ and urinary levels 29, 36 and 168 $IU\,l^{-1}$ respectively) (Figure 1). All three had poorly differentiated transitional cell carcinoma. One of these patients had metastatic bladder cancer, one extensive

Correspondence: J. McLoughlin.
Received 15 May 1990; and in revised form 11 December 1990.

**Figure 1** Urinary and Serum levels of β-HCG.

extravesical spread and one a locally resectable poorly differentiated malignancy. In this last patient the urinary levels of BhCG fell to within normal limits on estimation 2 weeks after resection of her bladder tumour.

A further three patients had elevations of BhCG in their serum alone (Figure 1). Two of these had poorly differentiated tumours (serum levels of 6 and 8 $IU/l^{-1}$ respectively) but the third had no sign of recurrence at the time of estimation (serum level 19 $IU\,l^{-1}$). This last patient had previously had regular well differentiated recurrences.

Immunoperoxidase staining of 28 sections and was performed. Twelve of these sections had evidence of well differentiated tumour present. Nine moderately well differentiated tumour and seven poorly differentiated transitional cell carcinomas present. Two of the specimens had evidence of carcinoma *in situ* (one of which also had a moderately well differentiated bladder tumour present in the same section). None of the tissues, including one of the patients with elevated urinary and plasma level of BhCG and the two patients who had tumour recurrence with raised serum levels of BhCG showed evidence of BhCG production. Applying the same technique to the controls (placental tissue) all stained positive for BhCG.

Follow up ranges from 11–17 months (mean 14). Of the patients with elevated serum and urinary levels of BhCG two failed to respond to chemotherapy and one has died from metastatic disease. Two of the three patients with elevation of serum levels alone were managed by chemotherapy and have to date remained clear of any recurrence. The third patient (who was noted to have an elevated BhCG level, but no recurrence) has developed further recurrence on two of three occasions following this time.

### Discussion

In this study, three of the 15 patients (20%) with poorly differentiated transitional cell carcinomas had both raised serum and urinary levels of BhCG. This compares to the report of Dexeus *et al.* (1986) who demonstrated that 38% of patients with advanced transitional cell carcinoma of the bladder had some degree of elevation of BhCG in their serum.

Using immunoperoxidase staining both Shah *et al.* (1986) and Rodenburg *et al.* (1985) observed BhCG production in poorly differentiated tumours and it has been suggested that a process of tumour de-differentiation was responsible for the production of BhCG (Shah *et al.*, 1986). Iles *et al.* (1987) however were able to demonstrate BhCG production in both neoplastic and non neoplastic cell lines suggesting that BhCG secretion is an innate property of bladder epithelium. It was on the basis of this that our investigation was performed, evaluating a range of tumours to assess whether levels were elevated in any cases of low grade tumours. Our results show that serum levels were not elevated in any case of well or moderately well differentiated bladder tumour.

Fukutami *et al.* (1983) demonstrated elevated urinary levels of BhCG in two of 10 patients with bladder carcinoma. A possible advantage of a urinary assay is that some tumours produce immunoreactive BhCG like substances (urinary gonadotrophin fragments) which, by virtue of subtle differences in their carbohydrate chains, may be rapidly cleared by the kidneys so that serum levels do not accumulate. It has been suggested that in urinary estimation of these substances may be a more reliable reflection of secretion (Nam *et al.*, 1990). In addition, in view of the fact that the bladder tumour is the direct site of production of the BhCG (Iles *et al.*, 1987; Shah *et al.*, 1986; Rodenburg *et al.*, 1985) it could be expected that the highest levels would be found in the urine. In this study however no patient was observed to have an elevated early morning urinary BhCG level in the presence of a serum level within the normal range.

In three cases serum levels alone were elevated. Two of these patients had grade 3 tumours (serum levels of 6 and 8 $IU\,l^{-1}$) present but one had no sign of recurrence at the time of estimation (serum level 19 $IU\,l^{-1}$). The significance of these results is unclear. In the two cases where tumour recurrence was noted and resected staining of the specimens revealed no evidence of BhCG production in the sections examined. Fukutami *et al.* (1983) suggested that greater sensitivity of assessing urinary BhCG levels could be obtained by estimating the 24 h urinary excretion of BhCG especially where serum levels are equivocal. The prospective nature of this study however precluded a retrospective estimation of 24 h urinary secretion in these patients as they had undergone tumour resection by the time their levels were available.

In the sections examined 12 had well, nine had moderately well and seven had poorly differentiated bladder tumour present. These sections included both of the patients who had elevated serum levels with tumour present (one patient with raised serum level had no sign of recurrence) but unfortunately only one of the three cases with raised serum and urinary levels of BhCG.

Shah *et al.* (1986) observed that the production of BhCG is often very localised and our negative result could reflect sampling error of the sections obtained. Alternatively the immunohistochemical method may be less sensitive than the polyclonal assay (which recognises the beta HCG core fragment). For example, techniques measuring B subunit core fragments have been shown to be of greater sensitivity than those estimating only free B subunits in patients with gynaecological malignancies (Cole *et al.*, 1988). The fact that the urinary levels fell rapidly following resection of the bladder tumour would suggest that the tumour was actually producing BhCG. Our positive controls were all positive suggesting that our lack of staining was not due to poor technique. In the other two cases, the patients had been referred for consideration for chemotherapy from other hospitals and no sections were available. (Formal histology reports were available and confirmed the presence of pooly differentiated transitional cell carcinoma of the bladder.)

From the results of this study BhCG would appear to be produced by only tumours of poorer differentiation and as such, measurement of BhCG as a tumour marker could be expected to have only a limited role in well or moderately well differentiated bladder tumours. Further studies, using large patient numbers, are necessary clarify this point.

### References

BRAUNSTEIN, G.D., VAITUKAITIS, J.C., CARBONE, P.P. & ROSS, G.T. (1973). Ectopic production of human chorionic gonadotrophin by neoplasms. *Ann. Intern. Med.*, **78**, 39.

COLE, L.A., WANG, Y., ELLIOT, M. & 4 others (1988). Urinary human chorionic gonadotrophin free Beta subunit and beta-core fragment: a new marker of gynaecological cancers. *Cancer Res.*, **48**, 1356.

DEXEUS, F., LOGOTHETIS, C., HOSSAN, E. & SAMUELS, M.L. (1986). Carcinoembryonic antigen and beta – human chorionic gonadotrophins as serum markers for advanced urothelial malignancies. *J. Urol.*, **136**, 403.

FUKUTANI, K., LIBBY, J.M., PANKO, W.B. & SCARDINO, P.T. (1983). Human chorionic gonadotrophin detected in urinary concentrates from patients with malignant tumours of the testes, prostate, bladder, ureter and kidney. *J. Urol.*, **129**, 74.

ILES, R.K., OLIVER, R.T.D., KITAU, M., WALKER, C. & CHARD, T. (1987). *In vitro* secretion of human chorionic gonadotrophin by bladder tumour cells. *Br. J. Cancer*, **55**, 623.

McMANUS, L.M., NAUGHTON, M.A. & MARTINEZ-HERNANDEZ, A.M. (1976). Human chorionic gonadotrophin in human neoplastic cells. *Cancer Res.*, **36**, 3476.

NAM, J.H., COLE, L.A., CHAMBERS, J.T. & SCHWARTZ, P.E. (1990). Urinary gonodotrophin fragment, a new tumour marker. *Gynaecol. Oncol.*, **36**, 383.

RODENBURG, C.J., NIEUWENHUYZEN KRUSEMAN, A.C., DE MAAKER, H.A., FIEUREN, E.J. & OOSTEROM, A.T. (1985). Immunohistochemical localisation and chromatographic characterisation of Human chorionic gonadotrophin in a bladder carcinoma. *Arch. Pathol. Lab. Med.*, **109**, 1046.

SHAH, V.M., NEWMAN, J., CROCKER, J. & 4 others (1986). Ectopic B-human chorionic gonadotrophin production by bladder urothelial neoplasia. *Arch. Pathol. Lab. Med.*, **110**, 107.